# Implementing Home Office Work at a Large Psychiatric University Hospital in Switzerland During the COVID-19 Pandemic: Field Report

**DOI:** 10.2196/28849

**Published:** 2021-09-01

**Authors:** Jana Sophia Krückl, Julian Moeller, Rainer Gaupp, Christoph E Meier, Carl Bénédict Roth, Undine Emmi Lang, Christian G Huber

**Affiliations:** 1 University Psychiatric Clinics Basel University of Basel Basel Switzerland; 2 Division of Clinical Psychology and Epidemiology Department of Psychology University of Basel Basel Switzerland

**Keywords:** home office, psychiatry, employees, mental health, depression, anxiety, stress factors, Patient Health Questionnaire, PHQ-2, General Anxiety Disorder, GAD-2, PHQ-D, COVID-19, pandemic

## Abstract

**Background:**

During the COVID-19 pandemic in 2020, psychiatric hospitals all over the world had to adapt their services to the prevailing governmental regulations. As a consequence, home office use and telepsychiatry boomed.

**Objective:**

The purpose of this study was to evaluate the potential of home office use, its adoption, and the association of home office use with employees’ mental health in a large psychiatric university hospital in Switzerland.

**Methods:**

We obtained and analyzed home office implementation and use data from the psychiatric university hospital’s information technology services. We also conducted a cross-sectional web-based survey to assess the employees’ attitudes toward the clinic’s crisis management during the COVID-19 pandemic in early 2020. Part of this web-based survey consisted of questions about home office use between March and June 2020, attitudes toward home office implementation, and mental health. Three mental health measures assessed depressive symptoms (Patient Health Questionnaire [PHQ]–2), anxiety (General Anxiety Disorder [GAD]–2), and stress factors (stress module of the PHQ-D); a cut-off score ≥3 was used for the PHQ-2 and GAD-2.

**Results:**

Of the 200 participating employees, 69 reported that they had worked from home at least partially (34.5%). Home office use differed significantly across professional groups (χ_16_^2^=72.72, *P*≤.001, n=200). Employees experienced neither depressive symptoms (mean 0.76, SD 1.14) nor anxiety (mean 0.70, SD 1.03). The employees reported minor psychosocial stressors (mean 2.83, SD 2.92). The number of reported stress factors varied significantly across groups with different levels of home office use (χ_4_^2^=9.72, *P*=.04).

**Conclusions:**

In general, home office implementation appears to be feasible for large psychiatric hospitals, however, it is not equally feasible for all professional groups. Professional groups that require personal contact with patients and technical or manual tasks must work onsite. Further evaluation of home office use in psychiatric hospitals up to the development of clinics that function merely online will follow in future research. The situation created by the COVID-19 pandemic served as a stepping stone to promote home office use and should be used to improve employees’ work–life balance, to save employers costs and foster other benefits.

## Introduction

Looking back on 2020, COVID-19 had the world firmly in its grip, with over 80 million confirmed cases and more than 1.8 million associated deaths [1]. After its first detection in China at the end of 2019, it spread rapidly around the globe. As a consequence, governments all over the world imposed major restrictions on the general population such as face masks requirements, social distancing requirements, and general lockdowns. In Switzerland, a national lockdown was declared shortly after the first confirmed cases in the country [2]; the general population was obliged to stay at home, shops were closed, and employees were urged to work from home, with only a few exceptions.

Researchers have reported numerous psychological effects of the pandemic [3-13]. Fear of transmission, isolation, unemployment, and economic recession were associated with increased distress [9,10], anxiety [9,10], and depression [4,9,10]. In particular, women [4,5,8,10], young people [7,8,10], individuals who lost their job [7,8,10], and individuals with a history of mental illness [10] seemed to suffer from negative consequences. Moreover, health professionals and mental health professionals reported increased distress during the pandemic, especially when facing COVID-19 infections at their workplace [3,5,6,14]. Accordingly, mental health services also face a rather high demand for psychiatric treatment during the ongoing pandemic [15,16]. However, it was difficult to offer treatment within the scope of the prevailing governmental measures (such as social distancing). This problem had to be solved, practically overnight, in psychiatric hospitals around the world. Traditional operating processes were adapted—new and especially safe approaches to offer psychiatric treatment while also preventing COVID-19 infections among patients and professionals.

Home office and telepsychiatry, the process of providing health care from a distance through technology [17], therefore, found their way into the daily routines of doctors, psychologists, and nurses in large psychiatric hospitals. Home office implementation has been associated with several benefits during the pandemic (ie, reduced COVID-19 infection risk due to reduced personal contact with co-workers and patients and less commuting) and beyond this extraordinary situation (ie, increased perceived autonomy in employees, higher job satisfaction, and less work–family conflicts [18-20]). Fadinger and Schymik [21] showed that home office use during the COVID-19 pandemic was associated with a lower infection risk and was less costly than confinement. However, home office use also exerts potentially detrimental effects on social relationships [18]. Moreover, home office work may not be feasible for all professional groups (eg, construction workers, nurses). Rutzer and Niggli [22] calculated a home office index that indicates the probability of being able to work from home (where 0 indicates that home office work is not possible, and 1 indicates that all work can be done from home). They found that the home office index differed between economic sectors as well as between professional groups. For the public health sector, the authors reported a home office index of 0.19 because there are many positions for which home office is not or minimally feasible. Because there are situations in which in-person treatments may be necessary, the implementation of home offices may be challenging for psychiatric hospitals.

However, numerous studies [23-27] since the 1990s have shown that telepsychiatry is comparable to in-person psychiatry (onsite psychiatric assessments and treatments) with respect to feasibility, validity, reliability of diagnoses, therapeutic alliance, and patient satisfaction, and doctor satisfaction. In addition, telepsychiatry increases accessibility for people living in rural areas, saves commuting time, and reduces costs [28]. However, there are also some challenges. First, certain technical prerequisites (ie, suitable devices for patients and health care professionals, and a stable internet connection) are required. Second, data security has to be ensured. Third, telepsychiatry may not be appropriate for certain populations (eg, suicidal or involuntarily treated patients and patients who struggle with navigating web-based platforms) [28].

Regarding these challenges, the implementation of telepsychiatry is an extremely complex and challenging process for mental health professionals, in general, and for large psychiatric hospitals, in particular. Before the COVID-19 pandemic, web-based treatment was not widely used in psychiatric hospitals in Switzerland; onsite treatment was the standard. However, the pandemic “has served as a catalyst for the rapid implementation and acceptance of telemental health” [28] as an effective option to deliver mental health services. Telepsychiatry (and home offices) suddenly became an integral part of work in psychiatric hospitals. However, the question arises—how will large psychiatric hospitals successfully implement home offices during the COVID-19 pandemic? We explored this issue by investigating the following research questions: How did home office use change over the course of the year 2020? Which employees were able to work in home office? How did the implementation of home office work from the employees’ viewpoint? Is home office use associated with the mental health of employees?

## Methods

### Background

The first case of COVID-19 in Switzerland was confirmed on February 25, 2020, and the first case in Basel was confirmed on February 27, 2020. Shortly afterward, on March 16, the Federal Council simultaneously declared extraordinary circumstances and a national lockdown [2,29]. Subsequently, the management board of the Psychiatric University Clinics Basel (UPK) requested that all employees for whom it was possible work from home. On June 19, 2020, the Federal Council eased the restrictions and ended the national status of extraordinary circumstances [2]. In autumn, the number of COVID-19 cases in Switzerland rose again, which led to renewed restrictions. On October 19, 2020, the Federal Council, therefore, recommended that employees work from home whenever possible [30]. These restrictions remained in place for the rest of the year and beyond. In 2020, a total of 8 patients with COVID-19 were treated at UPK.

### Research Design

We aimed to evaluate the potential of home office use, actual home office use, and the association of home office use with employees’ mental health for the staff from a large psychiatric university hospital (>1200 employees) in Switzerland. Background information about home office implementation and use were gathered by the hospital’s chief information officer (CM), and web-based survey data were collected as part of a retrospective analysis to assess the employees’ attitudes toward the clinic’s crisis management during the COVID-19 pandemic in early 2020. The cross-sectional web-based survey was mandated by the management board of UPK as a consequence of the far-reaching policies and extensive home office implementation in March 2020.

### Participants and Procedures

Home office users had access to the hospital’s home office environment (ie, their desktop and preinstalled apps) through Citrix Workspace (Citrix Systems Inc, 2020), with access from a large range of end devices with a broad selection of supported operating systems. This infrastructure had already been put into place before the pandemic but had only been used by a very limited number of employees. The email service was provided by internal Office Outlook servers (Windows 10; Microsoft Inc). In addition, a webmail service offered flexible email checking. Zoom (Zoom Video Communications Inc) was used for videoconferences. It was introduced at UPK in March 2020 to ensure efficient exchange between teams and individual employees and to provide a platform for telepsychiatry.

For the web-based survey, we estimated the required sample size using G*Power (version 3.1). We assumed medium effect sizes (*f*=0.25 [31]), α=.05, and power 0.8 [32]; the required sample size was 196. Based on expected attrition, we included 252 persons in the web-based survey. Because data were collected as part of a retrospective survey to assess crisis management, professional groups with a direct and significant effect on crisis management were included in the study: members of the crisis management group (n=23), supervising physicians, psychologists and nurses (n=93), link nurses (n=28), and employees of the ward established for COVID-19–positive patients (n=8). Link nurses are responsible for hospital hygiene on their division; they connect their division to the authorized representative for hospital hygiene of the canton Basel-City. In addition, a representative sample (with respect to profession, organizational unit, and years of professional experience) was randomly selected (n=100) from all other employees of UPK Basel. Exclusion criteria were employees with a small workload (<50%) and employees such as interns, medical student assistants, or without clinical or administrative duties. We assumed that these employees had not sufficiently been affected by the hospital’s clinical crisis management.

**Figure 1:**
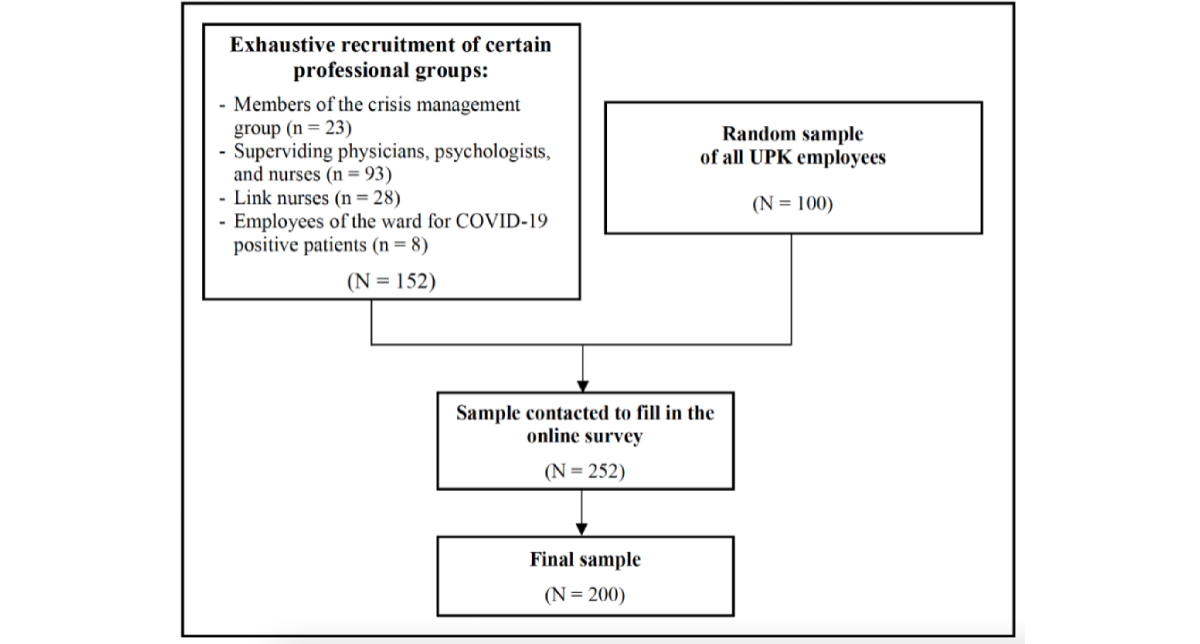
Sample composition of the web-based survey. UPK: Psychiatric University Clinics Basel.

Employees (n=252) were asked by email on June 10, 2020 to fill in the web-based survey. They received a reminder 7 days later and on June 26, 2020, which was 3 days before the assessment phase ended. A total of 200 employees (79.4% of the 252 initially approached employees) completed the web-based survey (Figure 1). Employees did not receive any compensation for their participation in the study. Data were anonymized and stored on a local server of the department of Quality and Processes at UPK Basel. Participants agreed to the publication of anonymized data.

No ethics committee approval was necessary; at the request of the authors, the Ethics Committee of Northwestern and Central Switzerland confirmed that these analyses do not fall within the scope of the Human Research Act (article 2 paragraph 1 [33]) as they are not defined as research concerning human diseases or concerning the structure and function of the human body.

### Measurements

#### Home Office Use

Frequency and distribution of videoconferences on Zoom were retrieved from the administrator account of the hospital’s chief information officer (CM). Data were downloaded on January 15, 2021. The frequencies of videoconferences over the course of the year 2020 were included as a proxy for home office use. Due to data protection regulations, detailed information about access to the home office environment are deleted after 30 days at UPK; therefore, these data were not available.

The web-based survey (Multimedia Appendix 1) asked “”Do you work from home?” Response options were “Yes, always”; “Yes, partially”; “No, it is not possible for my position”; “No, I did not want to”; or “No, I was rejected to work from home.” Employees’ rated several statements that assessed their attitudes toward the home office implementation (eg, “I have the necessary IT infrastructure available at home.”) on a 5-point Likert scale from “strongly disagree” to “strongly agree.”

#### Depression

The Patient Health Questionnaire (PHQ)-2 [34] assesses main criteria of depressive disorders with 2 items: “little interest or pleasure in doing things” and “feeling down, depressed, or hopeless.” Participants were asked to rate the frequency of these symptoms over the previous 2 weeks on a 4-point Likert scale from 0 (not at all) to 3 (nearly every day). The sum ranges between 0 and 6, and scores ≥3 have been shown to reliably screen for a current depressive episode [34]. This brief version of the PHQ-8 shows comparable reliability and validity for screening [34-36].

#### Anxiety

The General Anxiety Disorder (GAD)–2 scale [34] contains the first 2 items of the GAD-7 to assess core criteria of general anxiety disorder; a score ≥ 3 has been identified as the optimal cut-off for screening purposes. The GAD-2 has been shown to be a similarly valid and reliable screening instrument for all anxiety disorders (such as panic, social anxiety, and posttraumatic stress disorder) compared to the GAD-7 [34,36,37].

#### Stress

The stress scale of the PHQ–D [38] consists of 10 items to assess common psychosocial stressors (eg, financial status, family relationships, work). Each item is rated on a scale from 0 to 2 (not bothered, bothered a little, bothered a lot) [39]. The sum score (between 0 and 20) represents level of experienced stress. A score of 0 represents no stress factors, whereas a score of 20 stands for heavily experienced stress factors. No valid cut-off score is currently available for this stress scale [40]. In this sample, the stress scale of the PHQ-D showed acceptable to good internal consistency (Cronbach =0.78) [41]. The German version has been found to be a valid, reliable, and well-accepted screening instrument [38].

### Demographic Information

Sociodemographic data, including gender, professional group, and workload, were collected.

### Statistical Analysis

Descriptive statistics are presented. Frequencies and percentages are given for nominal data, and for interval data (ie, the questionnaires about mental health), mean and standard deviation were calculated. We divided the sample, first, on the basis on professional groups (ie, doctors, psychologists, nurses, employees working in administration, and others). The category *others* consisted of employees who did not belong to any of the other groups (ie, trainees, housekeeping, social services, etc). Second, we categorized the sample by home office use responses (ie, “Yes, always”; “Yes, partially”; “No, it is not possible for my position”; “No, I did not want to”; and “No, I was rejected to work from home”). The distribution of participants among the 5 home office groups were compared across the 5 professional groups using the Fisher exact test because group sizes were small. Cramer *V* was calculated to estimate the effect size.

Due to the nature of sample structure (ie, small group sizes), nonparametric tests (namely, the Kruskal-Wallis test) were used for comparisons regarding psychological well-being across home office groups and across professional groups. Exact calculation of the Monte-Carlo significance was chosen because of the small group sizes. For the final analysis, because some groups were too small to reliably conduct posthoc analyses; therefore, we used the Mann-Whitney U test to compare the 2 groups only—affirmative (“Yes, always” and “Yes, partially”) and negative (“No, it is not possible for my position”; “No, I did not want to”; and “No, I was rejected to work from home”).

Statistical analyses were performed using SPSS Statistics for Mac OS (version 27.0; IBM Corp), and graphical analyses were conducted in Excel for Mac (version 16.45; Microsoft Inc). Given the exploratory nature of this study, outliers were included in all analyses and no correction for multiple testing was applied. For all analyses, 2-tailed tests were used, and a significance level at 5% was chosen. Missing values were excluded pairwise.

## Results

**Figure 2:**
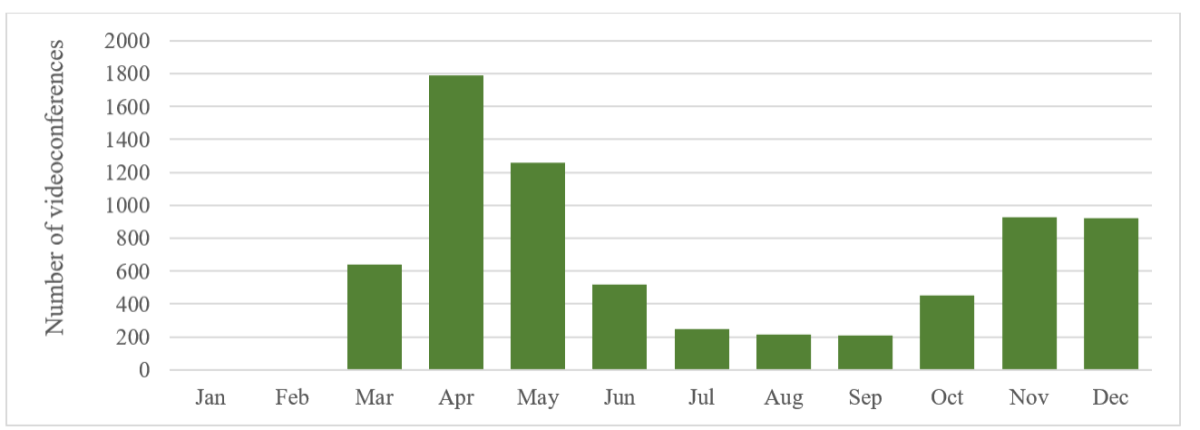
Distribution of videoconferences in 2020.

In total, 7173 videoconferences took place in 2020. More videoconferences were held in April 2020 (n=1788) than in any other month that year (Figure 2). In the month of April, the daily maximum was 125 videoconferences (on Tuesday April 20, 2020). Since then (over the months), the number of videoconferences has gradually decreased. For example, the daily maximum in June was 39 videoconferences (on Tuesday June 1 and Thursday June 3, 2020). In August, the daily maximum was 17 videoconferences (on Thursday August 12, 2020). The number of videoconferences again increased, to over 900 videoconferences per month in November and December. More detailed information (eg, videoconference participants or purpose) was not available due to data protection regulations.

Of the 200 web-based survey respondents, 115 (57.5%) were female. More than half of the sample (117/200, 58.5%) worked full-time (ie, level of employment between 90% and 100%), whereas the rest (n=83, 41.5%) worked between 50 and 89% (employees with a workload below 50% were excluded in advance).

The majority of employees continued to work at their original workspace (131/200, 65.5%) rather than working from home (69/200, 34.5%). For the majority of employees who were still working in person at the hospital, it was not possible for them to work from home because of their position (104/131, 79.4%). This seemed to be true for nurses in particular; 84.9% (62/73) reported that home office was not possible. In other professional groups, home office work seemed to be more feasible. Only 20% of employees in administration (6 out of 30) stated that home office was not possible. Most employees who worked from home worked part-time in person at their original working environment (61/69, 88%). Few employees worked full-time from home (8/69, 12%). Of 200 employees, 3 were denied the possibility of working from home (1.5%). The distribution across the 5 home office groups (Table 1) varied between the professional groups (χ_16_^2^=72.72, *P*≤.001, n=200). The effect size (Cramer *V*=.31) indicated a medium effect [42].

**Table 1:** Home office status (Did you work from home?) in the 5 professional groups during the COVID-19 pandemic in early 2020.

Responses	Doctors (N=42), n (%)	Psychologists (N=23), n (%)	Nurses (N=73), n (%)	Administration (N=30), n (%)	Others^a^ (N=32), n (%)	All (N=200), n (%)
Yes, always	1 (2.4)	1 (4.3)	0 (0.0)	3 (10.0)	3 (9.4)	8 (4.0)
Yes, partially	19 (45.2)	14 (60.9)	7 (9.6)	11 (36.7)	10 (31.3)	61 (30.5)
No, it was not possible for my position	17 (40.5)	6 (26.1)	62 (84.9)	6 (20.0)	13 (40.6)	104 (52.0)
No, I did not want to	4 (9.5)	1 (4.3)	4 (5.5)	10 (33.3)	5 (15.6)	24 (12.0)
No, I was rejected to work from home	1 (2.4)	1 (4.3)	0 (0.0)	0 (0.0)	1 (3.1)	3 (1.5)

^a^Employees who do not belong to any of the other categories (ie, trainees, housekeeping, social services, etc).

Employees who worked at least partially in home office (n=69) rated their work from home as a mainly positive experience. The majority of this subgroup had the necessary information technology infrastructure available at home (50/65, 76.9%) and promptly received a home office account from the information technology department (46/49, 93.9%). Most employees (55/66, 83.3%) had a quiet working space at home. Videoconferences via Zoom connected those working from home and those in the hospital. Zoom was seen as suitable for videoconferences or web-based therapy by 73.1% (106/145). Whereas the program was provided in time for 81.4% of the employees (131/161), many did not have sufficient equipment (eg, headset, webcam) for videoconferences (75/148, 50.7%). Almost half of the sample (83/170, 48.8%) reported that the help desk service of the information technology department was not available as usual.

Table 2 shows descriptive statistics of the 3 mental health measures (depression, anxiety, and psychosocial stressors). This sample seemed to experience only mild psychological distress, if at all. On average, employees reported minor psychosocial stressors (mean 2.83, SD 2.92). The only 2 exceptions were employees who had to work at home full-time and those who were rejected to work from home. Both groups reported a rather high number of psychosocial stressors (mean 5.13, SD 4.19 and mean 7.00, SD 5.20, respectively).

**Table 2:** Depression, anxiety, and psychosocial stressors for the whole sample and for the 5 home office groups separately.

			Depression^a^, mean (SD)		Anxiety^b^, mean (SD)		Stress factors^c^, mean (SD)
All			0.76 (1.14)		0.70 (1.03)		2.83 (2.92)
**Did you work from home?**							
	Yes, always	1.75 (1.91)		1.38 (1.77)		5.13 (4.19)	
	Yes, partially	0.69 (1.01)		0.53 (0.73)		2.12 (2.24)	
	No, it was not possible for my position	0.73 (1.11)		0.73 (1.14)		3.09 (3.08)	
	No, I did not want to	0.57 (0.99)		0.57 (0.73)		2.18 (1.94)	
	No, I was rejected to work from home	1.67 (2.08)		2.00 (0.00)		7.00 (5.20)	

^a^Assessed using Patient Health Questionnaire–2.

^b^Assessed using General Anxiety Disorder–2.

^c^Stress scale of Patient Health Questionnaire–D.

A Kruskal-Wallis H test revealed that the 5 home office groups differed concerning their reported number of stress factors (χ_4_^2^=9.72, *P*=.04). No significant differences were found for anxiety (χ_4_^2^=8.56, *P*=.07) or depression scores (χ_4_^2^=3.62, *P*=.47). A Mann-Whitney U test showed that the 2 groups (affirmative and negative) did not differ regarding reported stress factors (χ_4_^2^=3344.50, *P*=.17). The 5 professional groups also did not differ on any of the psychological scales (depression: χ_4_^2^=8.06, *P*=.08; anxiety: χ_4_^2^=3.17, *P*=.54; stress factors: χ_4_^2^=7.01, *P*=.13).

## Discussion

The aim of this field report was to describe the implementation of home office work for UPK staff in Basel, Switzerland during the COVID-19 pandemic. The national lockdown declared by the Swiss government in March 2020 boosted home office use, but home office use was not equally frequent in the different professional groups. UPK employees experienced no or only mild psychological distress during the current COVID-19 pandemic. Thus, the implementation of home office use for UPK staff can be seen as relatively successful; however, the broad implementation of home office in large psychiatric hospitals has to be viewed as a process that has just started [18].

COVID-19 and consequently declared governmental restrictions have provided a major impetus to telepsychiatry and home office implementation in Switzerland and all over the world [28]. At UPK, the use of the videoconferencing fluctuated along with governmental restrictions. In March 2020, the number of videoconferences increased sharply with the declaration of the national lockdown. Within days, the necessary technical requirements to offer videoconferences were set up by the hospital’s information technology department. Home office users grew from less than 100 to almost 400 employees, as every person had to work from home as long as the extraordinary circumstances [2,30] prevailed. The dramatically increasing capacity utilization, and lack of apps in the hospital’s home office environment were 2 major challenges in this time according to the hospital’s chief information officer. In June 2020, the Federal Council eased the restrictions [2], which led to a lower number of videoconferences—less than 250 per month—between July and September 2020. With the renewed rise of coronavirus infections in October 2020, the Federal Council again recommended that employees should work from home if possible [2,30]. The number of videoconferences, therefore, increased again at the end of this year, to almost 1000 videoconferences per month in the UPK Basel.

Interestingly, only one-third (69/200, 34.5%) of the UPK employees who responded to the web-based survey worked at least partially from home. The rest—approximately two-thirds—did not work from home at all. This ratio is in line with the home office index of 0.19 reported by Rutzer and Niggli [22] for the public health sector, where 19% of the positions or tasks can potentially be performed from home. Moreover, these findings also support the large differences between professional groups that have been reported [22]. Almost two-thirds of the psychologists (65.2%) answered that they work at least partially from home whereas only every tenth nurse did (9.6%). These percentages are in line with the home office indices reported by Rutzer and Niggli [22].

These large differences across professional groups correspond to work-related factors. According to Rutzer and Niggli [22], for employees in positions that require personal contact with clients or patients as well as mainly technical or manual tasks (eg, administering injections) must work in person (therefore, make home office impossible). Strategic, administrative, or creative tasks, on the other hand, can easily be completed from home (or any other place), which includes psychotherapeutic treatment [22]. As web-based assessments and treatment has been shown to be comparable to in-person appointments [23-25], home office use seems to be feasible even for large psychiatric university hospitals; however, as mentioned above, feasibility strongly differs between professional groups and may also depend on other factors (eg, inpatient vs outpatient services).

At UPK Basel, many of the employees in our sample did not want to work from home (24/200, 12.0%), especially those in administration (10/30, 33.3%). This choice belonged to the employees as the Federal Council only recommended—not required—that employees to work from home in March 2020. The attractiveness of home office use may therefore also depend on other factors (such as the employee’s personal living conditions). In spring 2020, more than 1200 employees of the UPK were challenged to adapt to novel working circumstances within a very short time. Nonetheless, employees rated home office implementation as mainly positive. They reported having the technical requirements as well as the necessary environmental conditions (such as a quiet working space). However, employees may have faced multiple (ie, not only work-related) challenges during these times. Suddenly, whole families were confined in their apartments; parents worked from home, and children had to be home-schooled. Several studies conducted before the COVID-19 pandemic reported the benefits of working from home (such as higher perceived autonomy, increased job satisfaction, and less work-family conflicts [18-20]), which might, however, have been reduced during these chaotic and insecure times.

Employees might have experienced heightened psychological distress as their daily routines suddenly dissolved and they had to take on new responsibilities (eg, home-schooling of their children). However, employees of the UPK reported no or only mild psychological distress in the web-based survey. In a meta-analysis, Batra et al [3] reported several risk factors for experiencing higher levels of depression and anxiety in health care workers (eg, being a nurse and being at risk of contact with COVID-19 patients). We found no differences between professional groups’ mental health measures; nurses did not experience heightened psychosocial stress in comparison to other professional groups in our findings. At UPK, only 8 patients with COVID-19 were treated. Employees might, therefore, have experience only mild anxiety and stress about possible COVID-19 infections.

The number of psychosocial stress factors differed across the home office groups but not between employees who worked from home and those who did not. Employees working full-time from home (n=8) as well as employees who were denied permission to work from home (n=3) seemed to experience substantially more stress factors than those experienced by other groups. These are 2 groups who reported some form of constraint (not being allowed to home office vs having to work in home office). This constraint may be seen in the framework of the locus of control theory [43]. An external locus of control means that a person believes that his or her life is controlled by factors outside his or her person (eg, by other people or fate) [43]. In previous studies [44-46], an external locus of control has been associated with mental health problems. These findings are in line with the results of our study that employees who did not have the choice of where to work seemed to experience more stress factors. Moreover, data analysis revealed a small group of employees who seemed to excessively suffer during the pandemic (n=8). Most of these employees perceived more psychological stress across several measures; however, there was no trend with respect to professional group, workload, or home office status. Although underlying reasons remain unknown, it is particularly important that employers maintain supportive relationships with their employees during exceptional times such as these. Detrimental work relationships, especially between employees, have been reported as negative consequences of home office use [18]. This risk has to be addressed during implementations of home office use. Nevertheless, most UPK employees reported no psychological distress during the insecure and challenging times of the COVID-19 pandemic.

The study had several limitations. First, a cross-sectional design might be neither appropriate for investigating the effects of the ongoing COVID-19 pandemic nor appropriate for investigating the time-consuming implementation of home office use. However, as exploratory analyses, the aim was to gain first impressions of how home office use was implemented in a large psychiatric hospital. Without question, further research is needed (eg, about maximizing the effectiveness of home office in psychiatric hospitals). Second, only a subgroup of the workforce at UPK participated in the web-based survey; however, all key employees were included (such as the crisis management group, link nurses, and employees of the newly established ward for COVID-19 positive patients), and with the random sample, it is assumed that the UPK workforce was well represented. Third, some videoconferences were possibly held onsite (instead of in home office) as meetings of more than 5 employees were prohibited for certain time periods. Videoconferences may, therefore, not reliably represent home office use. This limitation should be considered in future studies. Fourth, other variables that may have affected employees’ mental health (such as school-age children in the household, financial problems, etc) were not assessed. This limits generalizability. However, the focus of our findings was on home office implementation. Moreover, employees appeared to experience generally no or little psychological distress. It, therefore, can be assumed that the assessment of these variables would not have added substantial value to our findings. Fifth, the web-based survey was mandated by the management board of the UPK. This might have affected employees willingness to openly answer some questions (eg, questions about their mental health). As absolute anonymity was guaranteed, the bias is assumed to be negligible.

The situation created by the COVID-19 pandemic served as a stepping stone for home office use and telepsychiatry implementation in psychiatric hospitals all over the world. In large psychiatric hospitals, home office implementation is clearly feasible, and it will probably remain an inherent component of the working world. This shift offers numerous benefits for all involved, as long as the pitfalls of home office use are considered. However, the broad implementation of home office in psychiatry has just started. It is an ongoing process that requires further observation and research (eg, about the efficient use of home office in large psychiatric hospitals). Thus, the pandemic, in spite of its sudden appearance, will probably have long-term effects on our daily lives and on mental health care.

## Appendix

Multimedia Appendix 1 Web-based survey items: home office and information technology services.

## Abbreviations

GAD: General Anxiety Disorder
PHQ: Patient Health Questionnaire
UPK: Psychiatric University Clinics Basel

## References

[1] (2020). WHO coronavirus disease (COVID-19) dashboard. World Health Organization (WHO).

[2] Coronavirus: Massnahmen und Verordnungen. Bundesamt für Gesundheit (BAG).

[3] Batra K, Singh TP, Sharma M, Batra R, Schvaneveldt N (2020). Investigating the psychological impact of COVID-19 among healthcare workers: a meta-analysis. Int J Environ Res Public Health.

[4] Debowska A, Horeczy B, Boduszek D, Dolinski D (2020). A repeated cross-sectional survey assessing university students' stress, depression, anxiety, and suicidality in the early stages of the COVID-19 pandemic in Poland. Psychol Med.

[5] Barzilay R, Moore TM, Greenberg DM, DiDomenico GE, Brown LA, White LK, Gur RC, Gur RE (2020). Resilience, COVID-19-related stress, anxiety and depression during the pandemic in a large population enriched for healthcare providers. Transl Psychiatry.

[6] Zhang W, Wang K, Yin L, Zhao W, Xue Q, Peng M, Min B, Tian Q, Leng H, Du J, Chang H, Yang Y, Li W, Shangguan F, Yan T, Dong H, Han Y, Wang Y, Cosci F, Wang H (2020). Mental health and psychosocial problems of medical health workers during the COVID-19 epidemic in China. Psychother Psychosom.

[7] Every-Palmer S, Jenkins M, Gendall P, Hoek J, Beaglehole B, Bell C, Williman J, Rapsey C, Stanley J (2020). Psychological distress, anxiety, family violence, suicidality, and wellbeing in New Zealand during the COVID-19 lockdown: a cross-sectional study. PLoS One.

[8] Rodríguez-Rey Rocío, Garrido-Hernansaiz H, Collado S (2020). Psychological impact and associated factors during the initial stage of the coronavirus (COVID-19) pandemic among the general population in Spain. Front Psychol.

[9] Salari N, Hosseinian-Far A, Jalali R, Vaisi-Raygani A, Rasoulpoor S, Mohammadi M, Rasoulpoor S, Khaledi-Paveh B (2020). Prevalence of stress, anxiety, depression among the general population during the COVID-19 pandemic: a systematic review and meta-analysis. Global Health.

[10] Xiong J, Lipsitz O, Nasri F, Lui LM, Gill H, Phan L, Chen-Li D, Iacobucci M, Ho R, Majeed A, McIntyre RS (2020). Impact of COVID-19 pandemic on mental health in the general population: a systematic review. J Affect Disord.

[11] McIntyre RS, Lee Y (2020). Preventing suicide in the context of the COVID-19 pandemic. World Psychiatry.

[12] Mamun MA, Ullah I (2020). COVID-19 suicides in Pakistan, dying off not COVID-19 fear but poverty? - the forthcoming economic challenges for a developing country. Brain Behav Immun.

[13] Thakur V, Jain A (2020). COVID 2019-suicides: a global psychological pandemic. Brain Behav Immun.

[14] Lai J, Ma S, Wang Y, Cai Z, Hu J, Wei N, Wu J, Du H, Chen T, Li R, Tan H, Kang L, Yao L, Huang M, Wang H, Wang G, Liu Z, Hu S (2020). Factors associated with mental health outcomes among health care workers exposed to coronavirus disease 2019. JAMA Netw Open.

[15] Titov N, Staples L, Kayrouz R, Cross S, Karin E, Ryan K, Dear B, Nielssen O (2020). Rapid report: Early demand, profiles and concerns of mental health users during the coronavirus (COVID-19) pandemic. Internet Interv.

[16] Thome J, Deloyer J, Coogan AN, Bailey-Rodriguez D, da Cruz E Silva Odete A B, Faltraco F, Grima C, Gudjonsson SO, Hanon C, Hollý Martin, Joosten J, Karlsson I, Kelemen G, Korman M, Krysta K, Lichterman B, Loganovsky K, Marazziti D, Maraitou M, Mertens deWilmars S, Reunamen M, Rexhaj S, Sancaktar M, Sempere J, Tournier I, Weynant E, Vis C, Lebas M, Fond-Harmant L (2020). The impact of the early phase of the COVID-19 pandemic on mental-health services in Europe. World J Biol Psychiatry.

[17] What is Telepsychiatry?. American Psychiatric Association.

[18] Gajendran RS, Harrison DA (2007). The good, the bad, and the unknown about telecommuting: meta-analysis of psychological mediators and individual consequences. J Appl Psychol.

[19] Hill E, Ferris M, Märtinson V (2003). Does it matter where you work? a comparison of how three work venues (traditional office, virtual office, and home office) influence aspects of work and personal/family life. J Vocat Behav.

[20] Baruch Y (2002). Teleworking: benefits and pitfalls as perceived by professionals and managers. New Technol Work Employ.

[21] Fadinger H, Schymik J (2020). The costs and benefits of home office during the COVID-19 pandemic: evidence from infections and an input-output model for Germany. COVID Economics.

[22] Rutzer C, Niggli M Corona-Lockdown und Homeoffice in der Schweiz. Universität Basel Center for International Economics and Business.

[23] Hyler SE, Gangure DP, Batchelder ST (2005). Can telepsychiatry replace in-person psychiatric assessments? a review and meta-analysis of comparison studies. CNS Spectr.

[24] García-Lizana F, Muñoz-Mayorga I (2010). What about telepsychiatry? a systematic review. Prim Care Companion J Clin Psychiatry.

[25] Hilty DM, Ferrer DC, Parish MB, Johnston B, Callahan EJ, Yellowlees PM (2013). The effectiveness of telemental health: a 2013 review. Telemed J E Health.

[26] Berryhill MB, Culmer N, Williams N, Halli-Tierney A, Betancourt A, Roberts H, King M (2019). Videoconferencing psychotherapy and depression: a systematic review. Telemed J E Health.

[27] Berger T (2017). The therapeutic alliance in internet interventions: a narrative review and suggestions for future research. Psychother Res.

[28] Madigan S, Racine N, Cooke JE, Korczak DJ (2021). COVID-19 and telemental health: benefits, challenges, and future directions. Canadian Psychology.

[29] (2020). Coronavirus: Bundesrat erklärt die «ausserordentliche Lage» und verschärft die Massnahmen. Bundesamt für Gesundheit (BAG).

[30] Lockerungen und Verschärfungen der nationalen Massnahmen. Bundesamt für Gesundheit (BAG).

[31] Cohen J (1988). Statistical Power Analysis for the Behavioral Sciences 2nd ed.

[32] Bühner M, Ziegler M (2009). Statistik für Psychologen und Sozialwissenschaftler.

[33] Federal act on research involving human beings. Fedlex.

[34] Kroenke K, Spitzer RL, Williams JB, Löwe Bernd (2009). An ultra-brief screening scale for anxiety and depression: the PHQ-4. Psychosomatics.

[35] Löwe Bernd, Kroenke K, Gräfe Kerstin (2005). Detecting and monitoring depression with a two-item questionnaire (PHQ-2). J Psychosom Res.

[36] Kroenke K, Spitzer RL, Williams JBW, Löwe Bernd (2010). The patient health questionnaire somatic, anxiety, and depressive symptom scales: a systematic review. Gen Hosp Psychiatry.

[37] Kroenke K, Spitzer RL, Williams JB, Monahan PO, Löwe Bernd (2007). Anxiety disorders in primary care: prevalence, impairment, comorbidity, and detection. Ann Intern Med.

[38] Gräfe K, Zipfel S, Herzog W, Löwe B (2004). Screening psychischer Störungen mit dem “Gesundheitsfragebogen für Patienten (PHQ-D)“. Diagnostica.

[39] Klapow J, Kroenke K, Horton T, Schmidt S, Spitzer R, Williams JBW (2002). Psychological disorders and distress in older primary care patients: a comparison of older and younger samples. Psychosom Med.

[40] Beutel TF, Zwerenz R, Michal M (2018). Psychosocial stress impairs health behavior in patients with mental disorders. BMC Psychiatry.

[41] Blanz M (2021). Forschungsmethoden und Statistik für die Soziale Arbeit: Grundlagen und Anwendungen.

[42] Ellis P (2010). The Essential Guide to Effect Sizes: Statistical Power, Meta-analysis, and the Interpretation of Research Results.

[43] Rotter JB (1966). Generalized expectancies for internal versus external control of reinforcement. Psychol Monogr.

[44] Hovenkamp-Hermelink JH, Jeronimus BF, van der Veen DC, Spinhoven P, Penninx BW, Schoevers RA, Riese H (2019). Differential associations of locus of control with anxiety, depression and life-events: a five-wave, nine-year study to test stability and change. J Affect Disord.

[45] Wiersma JE, van Oppen P, van Schaik DJF, van der Does AJW, Beekman ATF, Penninx BWJH (2011). Psychological characteristics of chronic depression: a longitudinal cohort study. J Clin Psychiatry.

[46] Struijs SY, Groenewold NA, Oude Voshaar RC, de Jonge P (2013). Cognitive vulnerability differentially predicts symptom dimensions of depression. J Affect Disord.

