# Supplementary material for: Implementing Home Office Work at a Large Psychiatric University Hospital in Switzerland During the COVID-19 Pandemic: Field Report
Source: JMIR Ment Health. 2021 Sep 1;8(9):e28849. doi: 10.2196/28849 (PMC8412137; doi:10.2196/28849)
Supplement: Multimedia Appendix 1 [file mental_v8i9e28849_app1.docx]

# Multimedia Appendix

**Online survey: items concerning HO and IT services.**

|  |  | **Strongly disagree** | **Disagree** | **Neutral** | **Agree** | **Strongly agree** |
| --- | --- | --- | --- | --- | --- | --- |
|  |  |  |  |  |  |  |
| **Home Office (HO)** | I have the necessary IT infrastructure available at home. |  |  |  |  |  |
|  | I promptly received a home office account by the IT department. |  |  |  |  |  |
|  | I have a quiet working space at home. |  |  |  |  |  |
| **IT Services** | Zoom® is suitable for video conferences and/or online therapy. |  |  |  |  |  |
|  | The program ZOOM® was provided in time by the IT department. |  |  |  |  |  |
|  | I have sufficient equipment for video conferences (e.g., headset, webcam). |  |  |  |  |  |
|  | The help desk service of the IT department was available all the time. |  |  |  |  |  |

|  |  |  |  |  |
| --- | --- | --- | --- | --- |
| 0.76 | (1.14) |  | 0.70 | (1.03) |
